# Estimating chronological age through learning local and global features of panoramic radiographs in the Korean population

**DOI:** 10.1038/s41598-023-48960-2

**Published:** 2023-12-09

**Authors:** Han-Gyeol Yeom, Byung-Do Lee, Wan Lee, Taehan Lee, Jong Pil Yun

**Affiliations:** 1 Department of Oral and Maxillofacial Radiology and Wonkwang Dental Research Institute, College of Dentistry, Wonkwang University, Iksan, Republic of Korea; 2 AI Research Center for Manufacturing Systems (AIMS), Korea Institute of Industrial Technology (KITECH), Daegu, 42994 Republic of Korea; 3 University of Science and Technology, Daegu, Republic of Korea

**Keywords:** Medical research, Medical imaging, Dentistry, Dental radiology, Forensic dentistry

## Abstract

This study suggests a hybrid method based on ResNet50 and vision transformer (ViT) in an age estimation model. To this end, panoramic radiographs are used for learning by considering both local features and global information, which is important in estimating age. Transverse and longitudinal panoramic images of 9663 patients were selected (4774 males and 4889 females with a mean age of 39 years and 3 months). To compare ResNet50, ViT, and the hybrid model, the mean absolute error, mean square error, root mean square error, and coefficient of determination (R^2^) were used as metrics. The results confirmed that the age estimation model designed using the hybrid method performed better than those using only ResNet50 or ViT. The estimation is highly accurate for young people at an age with distinct growth characteristics. When examining the basis for age estimation in the hybrid model through attention rollout, the proposed model used logical and important factors rather than relying on unclear elements as the basis for age estimation.

## Introduction

In the forensic field, age estimation is a crucial step in biological identification. Age estimation is required for identifying the deceased, and for living people, particularly children, and adolescents, it is essential for answering numerous legal questions and resolving civil and judicial issues^1,2^.

Numerous techniques are available for estimating age using various body components. Several studies have focused on the connection between epiphyseal closure and age^3,4^. Many factors including sex, genetics, and geography are related to epiphyseal fusion^3,5^. However, because of incomplete skeletal development, the bone age assessment method is usually adopted to evaluate immature individuals^6^.

Evaluation of dental age using radiographic tooth development and tooth eruption sequences is more accurate than other methods^7,8^. Because tooth and dental tissue is largely genetically formed and is less susceptible to environmental and dietary influences, there is less deformation caused by external chemical and physical damage^2,3,7^.

Many attempts have been made to create standards for age estimation using human interpretations of dental radiological images. The Demirjian technique, which is the most common method, divides teeth into eight categories (A–H) based on their maturity and degree of calcification^9^. Willems et al. modified this method and provided a new scoring method that allows direct conversion from classification to age^10^. Cameriere established a European formula by gauging the open apices of seven permanent teeth in the left mandible on panoramic radiographs^11^. However, these methods have a certain degree of subjectivity, leading to a relatively high level of personal error, and their application requires adequate experience to minimize errors^12^. Furthermore, there are fundamental limitations to its applicability in young subjects. For age estimation in adults, a feasible approach involves proposing the calculation of the pulp-tooth area ratio calculation^13,14^. Another recommended method is the pulp/tooth width–length ratio calculation^15^. While the utilization of population-specific formulae was advised, the incorporation of data from individuals across diverse population groups into the same analysis was not discouraged^16^.

Machine learning, the cornerstone of artificial intelligence, enables more precise and effective dental age prediction^12,17,18^. Tao and Galibourg applied machine learning to the Demirjian and Willams methods for dental age estimation^17,18^, and Shihui et al.^12^ used the Cameriere method. Most studies related to age estimation use convolutional neural network (CNN)-based models^19–22^. Such models learn local features well because of the convolution filter operation but do not learn global information well. This problem can be solved by learning local features and global information using a vision transformer (ViT)^23^. In addition, the hybrid method, which uses the feature map extracted from the CNN-based model as input to the ViT model, displays better image classification performance than using each model alone^23^. Therefore, we adopted a hybrid method to design an age estimation model because learning by considering both local features that distinguish fine differences in teeth or periodontal region, and global information that better understands the overall oral structure is important for estimating age.

This study constructed an age estimation model using a hybrid method of the ResNet50 and ViT models. Subsequently, we confirm whether the model performs better so that it can be used effectively in clinical field.

## Materials and methods

### Data set and image pre-processing

We collected transverse and longitudinal panoramic images of patients who visited the Daejeon Wonkwang University Dental Hospital. All panoramic images obtained between January 2020 and June 2021 were randomly selected. When multiple images were available for a patient, the initially obtained image was chosen. Exclusion criteria involved images with unsuitable image quality, as determined by the consensus of three oral and maxillofacial radiologists.

A total of 9663 panoramic radiographs were selected (4774 males and 4889 females; mean age 39 years 3 months). Panoramic images were obtained using three different panoramic machines: Promax® (Planmeca OY, Helsinki, Finland), PCH-2500® (Vatech, Hwaseong, Korea), and CS 8100 3D® (Carestream, Rochester, New York, USA). Images were extracted using the DICOM format.

The age of the acquired data ranged from 3 years 4 months to 79 years 1 month (Table 1). Because the amount of data for each age group differed and may adversely affect the results if used randomly, the amount for each age group was divided by a 6:2:2 ratio to balance the data among the training, validation, and test sets. Thus, 5861 training, 1916 validation, and 1886 test data were used.

**Table 1:** Number of panoramic radiographs by age.

Age (years)	No. of panoramic radiographs
3–9	892
10–19	999
20–29	2029
30–39	1067
40–49	1134
50–59	1609
60–69	1285
70–79	648
Total	9663

The edge of the image was cropped to focus on the meaningful region and filled with zero padding around the image. Additionally, because the image sizes obtained from the two devices were different (2868 × 1504 pixels and 2828 × 1376 pixels), the images were resized to the same size (384 × 384 pixels) for batch learning and to improve learning speed.

To learn more effectively with the acquired data, augmentation techniques using normalization, horizontal flip with a probability of 0.5, and color jitter were applied to the training set.

### Architecture of deep-learning model

We used two types of age estimation models. The first is ResNet, a well-known CNN-based model which has been used as a feature extractor in many studies related to age prediction^24,25^. ResNet can build deep layers by solving the gradient vanishing problem through residual learning using skip connection^26^. However, because the model has a locality inductive bias, relatively less global information is learned than the local features. The other is the ViT^23^, which uses a transformer^27^ encoder and lacks inductive bias compared with CNN-based models. However, by performing pre-training on large datasets such as ImageNet21k, it overcomes structural limitations. The model has a wide range of attention distances that can learn the global information and local features. Additionally, the model also exhibited better classification performance than CNN-based models. Using the strengths of these two models, we propose an age prediction model based on ResNet50-ViT^23^, a hybrid method that can effectively learn the global information which better understands the overall oral structure and local features that distinguish fine differences in teeth or periodontal region.

**Figure 1:**
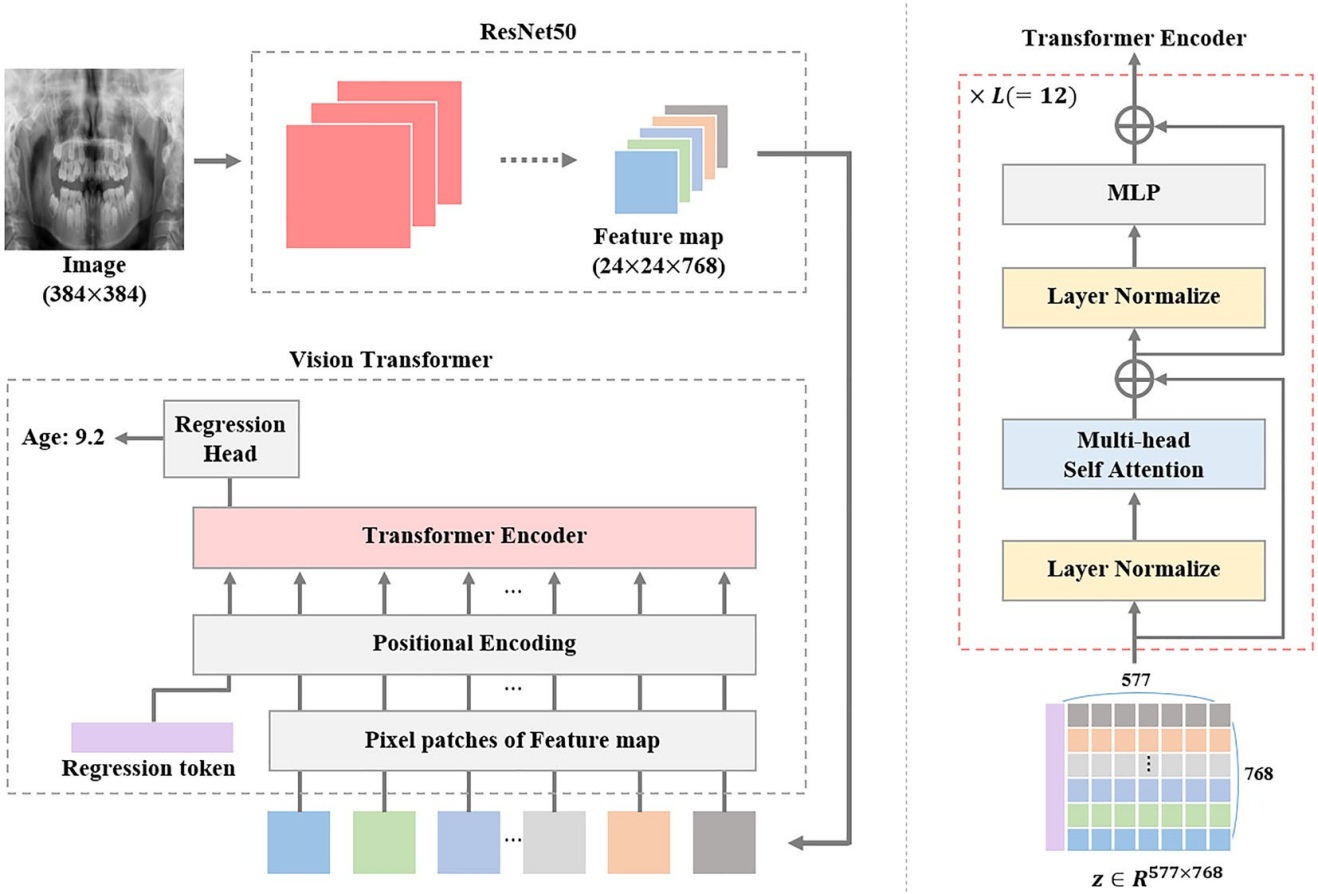
Proposed architecture of age estimation model.

The overall architecture of the proposed model is presented in Fig. 1. The feature map $\mathbf{x}\in {R}^{H\times W\times C}$ extracted by placing the panoramic image into ResNet50 was used as the input patch for the transformer, where $(H, W)$ are the height and width of the feature map, respectively, and $C$ is the number of channels. We define $HW(=N)$ as the total number of patches because each pixel in the feature map is considered a separate patch.

To retain the positional information of the extracted feature map, we add a trainable positional encoding ${{\varvec{x}}}_{{\varvec{p}}{\varvec{o}}{\varvec{s}}}\in {R}^{(N+1)\times C}$ to the sequence of feature patch: ${\varvec{z}}=[{x}_{reg};{x}_{1};{x}_{2};...;{x}_{N}]+{{\varvec{x}}}_{{\varvec{p}}{\varvec{o}}{\varvec{s}}}$ where ${x}_{reg}\in {R}^{C}$ is trainable regression token, and ${x}_{i}$ is $i$ th patch of the feature map.

Then, the ${\varvec{z}}$ is entered into the transformer encoder blocks composed of the layer norm (LN)^28^, multi-head self-attention (MSA)^23,27^, and multilayer perceptron (MLP), which contains two linear layers with a Gaussian Error Linear Unit (GELU) function. The transformer encoder process is as follows:

$$\overline{z}^{l} = MSA\left( {LN\left( {z^{l} } \right)} \right) + z^{l} ,{ }l = 1,2, \ldots ,L$$

$$z^{l + 1} = MLP\left( {LN\left( {\overline{z}^{l} } \right)} \right) + \overline{z}^{l} ,{ }l = 1,2, \ldots ,L$$

where $l$ denotes the $l$ th transformer encoder block. Finally, we estimated the age from the regression head using ${z}_{reg}^{L+1}$.

### Learning details

To train the model efficiently, we employed transfer learning, which aids in overcoming weak inductive bias and improving accuracy. That is, we initially set the parameters of the models using weights pre-trained using ImageNet21k and then fine-tuned using our panoramic-image dataset. The models were trained with a stochastic gradient descent (SGD) optimizer with a momentum of 0.9, learning rate of 0.01, and batch size of 16; for 100 epochs, the objective function was the mean absolute error (MAE). After training on the training set at every epoch, we performed an evaluation using the validation set. When the training was completed, the weight parameter of the model with the best MAE in the validation set was stored.

### Ethical approval and informed consent

This study was conducted in accordance with the guidelines of the World Medical Association Helsinki Declaration for Biomedical Research Involving Human Subjects. It was approved by the Institutional Review Board of Daejeon Dental Hospital, Wonkwang University (W2304/003-001). Owing to the non-interventional retrospective design of this study and because all data were analyzed anonymously, the IRB waived the need for individual informed consent, either written or verbal, from the participants.

## Results

**Figure 2:**
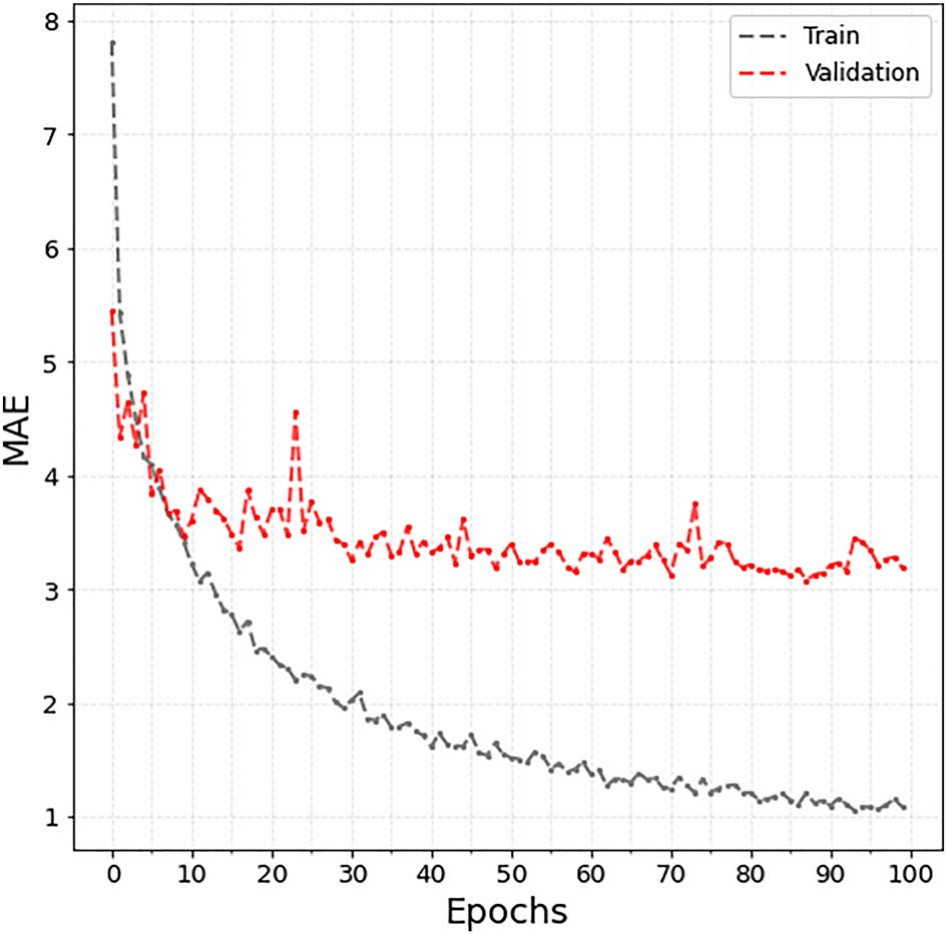
Loss plot for training and validation set.

**Figure 3:**
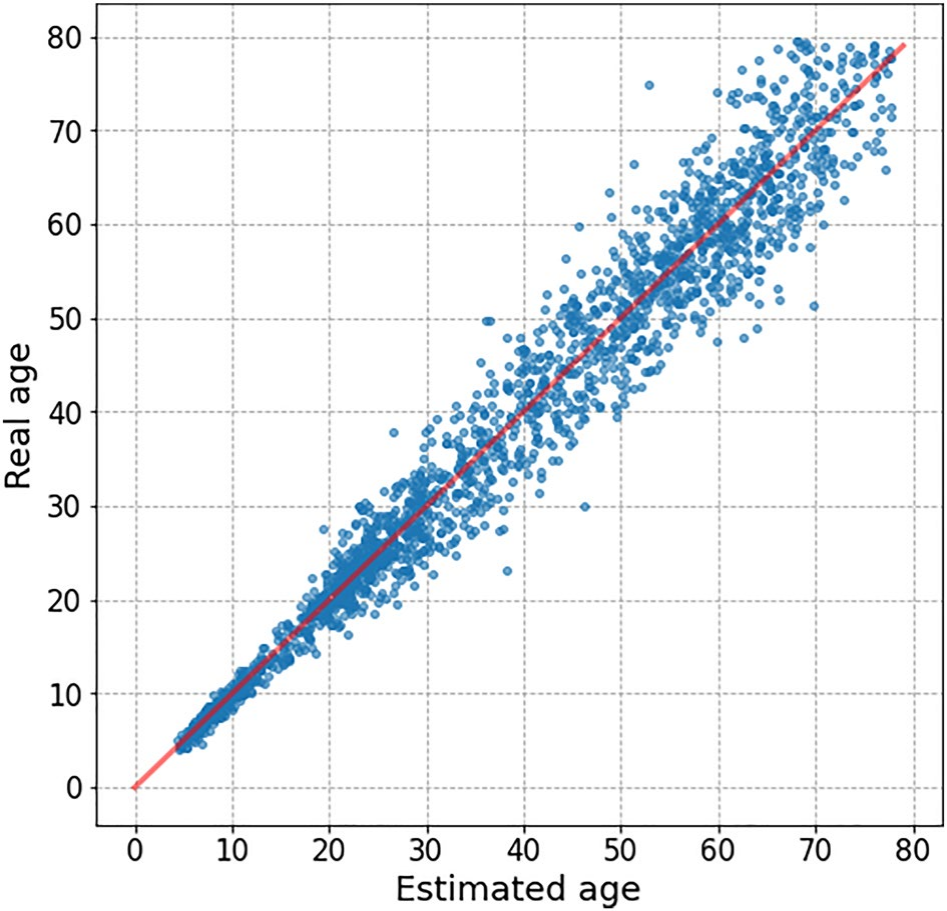
Scatter plot for test results.

The losses for the training and validation sets at each epoch are plotted in Fig. 2. The model with the best MAE was selected from the validation set for testing. As a result, the MAE of the hybrid age estimation model for the 1886 test data was 2 years and 11 months (2.95 years). A scatter plot of the estimated and chronologic ages is presented in Fig. 3.

In addition, as confirmed in Table 2, the age estimation model designed using the hybrid method performs better than that designed using only ResNet50 or ViT. For comparison, the MAE, mean square error (MSE), root mean square error (RMSE), and coefficient of determination (${\mathrm{R}}^{2}$) were used as the evaluation metrics.

$$MAE = \frac{1}{N}\mathop \sum \limits_{i = 1}^{N} \left| {\hat{y}_{i} - y_{i} } \right|,$$

$$MSE = \frac{1}{N}\mathop \sum \limits_{i = 1}^{N} \left( {\hat{y}_{i} - y_{i} } \right)^{2} ,$$

$$RMSE = \sqrt {\frac{1}{N}\mathop \sum \limits_{i = 1}^{N} \left( {\hat{y}_{i} - y_{i} } \right)^{2} } ,$$

$$R^{2} = 1 - \frac{{\mathop \sum \nolimits_{i = 1}^{N} \left( {\hat{y}_{i} - y_{i} } \right)^{2} }}{{\mathop \sum \nolimits_{i = 1}^{N} \left( {\overline{y} - y_{i} } \right)^{2} }},$$

where $N$ is cardinality of dataset, ${\widehat{y}}_{i}$ and ${y}_{i}$ denote estimated and chronologic age for $i$-$th$ data, $\overline{y }$ represents mean of the chronologic age $\frac{1}{N}\sum_{i=1}^{N}{y}_{i}$.

**Table 2:** Model performance.

	ResNet50	ViT	Hybrid
MAE	3.20	4.09	2.95
MSE	18.59	32.57	16.76
RMSE	4.31	5.70	4.09
${\mathrm{R}}^{2}$	0.95	0.92	0.95

*MAE* mean absolute error, *MSE* mean square error, *RMSE* root mean square error, ${R}^{2}:$ coefficient of determination.

**Figure 4:**
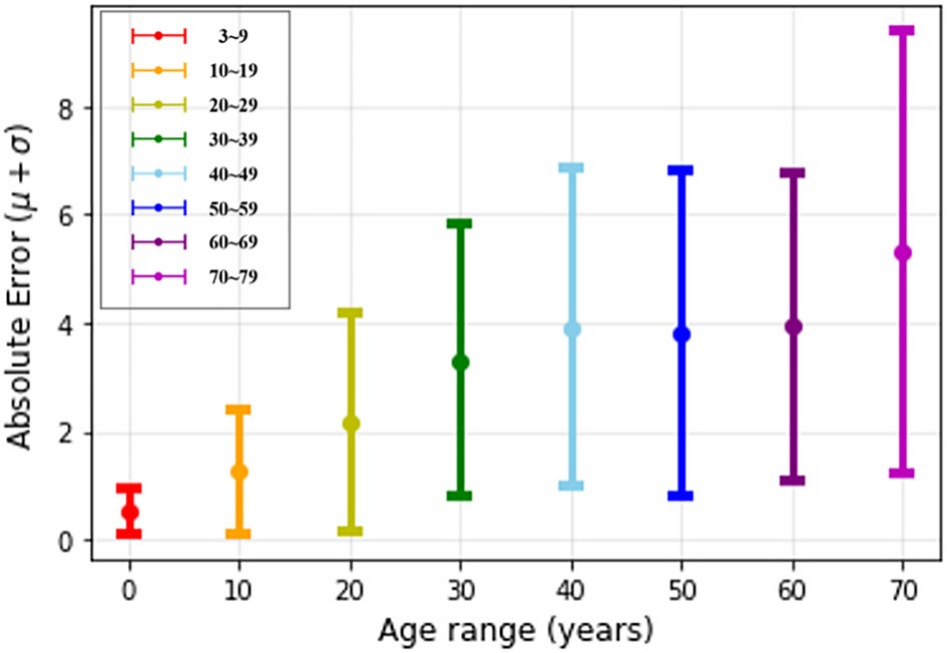
Absolute error by age range.

As illustrated in Fig. 4, the estimation is highly accurate for young people at an age with distinct growth characteristics. However, as aging progresses, the error tends to increase. Detailed information about performance is contained in Table 3.

**Table 3:** The performance of hybrid method by age range.

Age (years)	MAE	MSE	RMSE
3–9	0.52	0.45	0.67
10–19	1.27	2.96	1.72
20–29	2.17	8.73	2.95
30–39	3.30	17.21	4.14
40–49	3.92	24.12	4.91
50–59	3.81	23.54	4.85
60–69	3.94	23.69	4.86
70–79	5.30	44.85	6.69

*MAE* mean absolute error, *MSE* mean square error, *RMSE* root mean square error.

Finally, we used attention rollout^29^, which is a suitable method for visualization in a transformer-based structure, to analyze the results of the model.

## Discussion

Compared to previous age estimation studies that did not employ deep learning^15^, significant improvements were observed in both MAE and RMSE values across all network models (ResNet50, ViT, and Hybrid).

The structure of the oral maxillofacial region can be observed on a single two-dimensional image using panoramic radiography. As the principle of such radiography is the combination of tomography and scanning, only the structure located in the image layer can be clearly obtained, interpreted, and have diagnostic value^30^. Therefore, even if we take a panoramic radiograph of the same patient, extremely different images can be obtained depending on the positioning of the patient, type of equipment, and patient management skill of the radiographer. Therefore, deep-learning models that are more meaningful and can be used in clinical practice should be constructed through training with panoramic radiographs obtained by multiple radiographers using multiple pieces of equipment. We used images obtained using three pieces of equipment by more than 15 radiographers. In addition, using only Korean data (approximately 10,000 data), it was possible to effectively learn the differences by age by minimizing the differences in racial factors.

Because most studies related to age prediction use CNN-based models, local features were learned well, but global information was not. This study proposes an age prediction model that learns such information and local features via a hybrid model using a CNN-based ResNet50 and transformer-based ViT. The results confirmed that the proposed model effectively predicted age by performing better than ResNet50 or ViT (Table 2).

**Figure 5:**
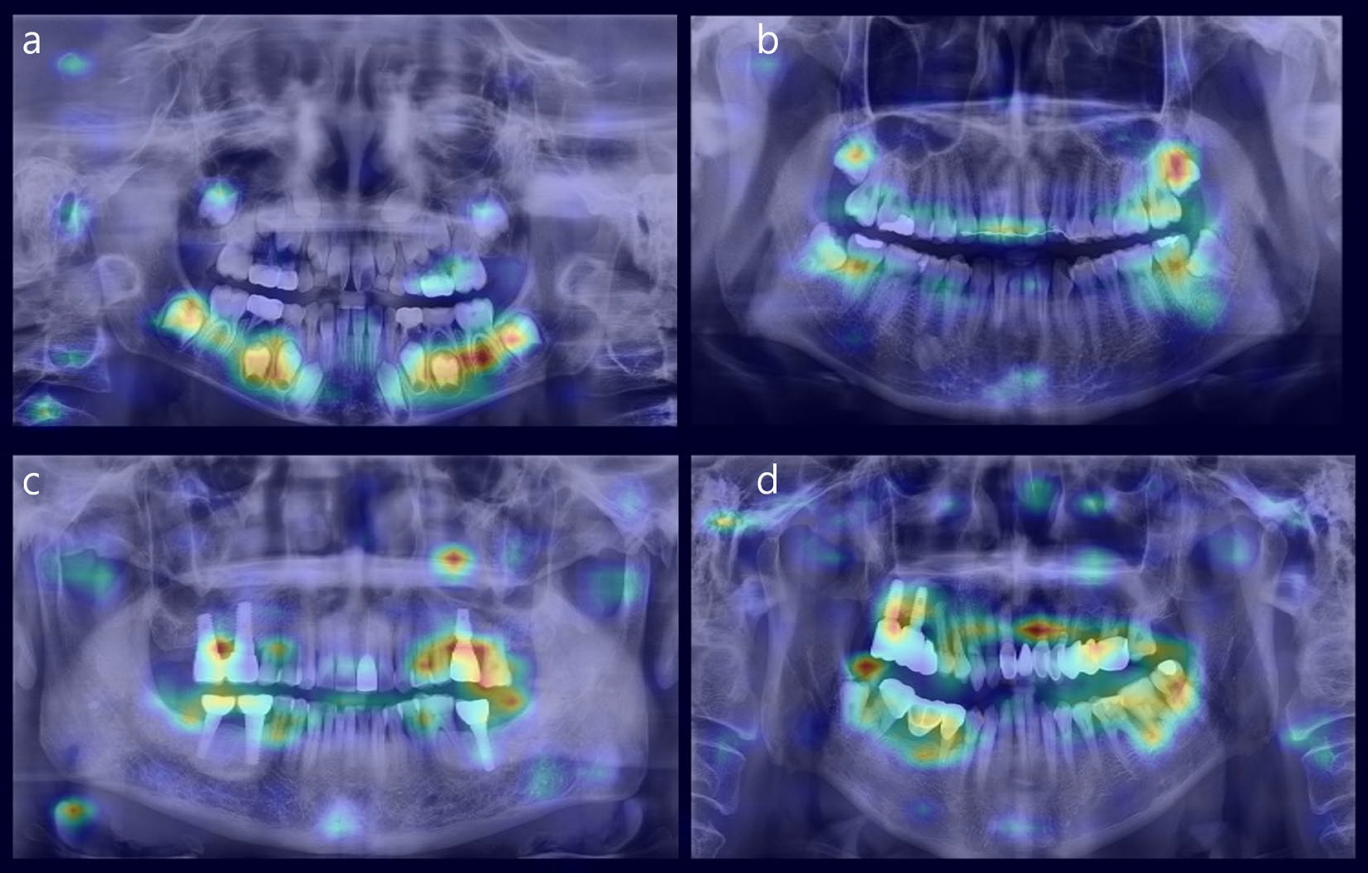
Example panoramic images of heatmap visualization by attention rollout (**a**) patient aged 7, (**b**) patient aged 19, (**c**) patient aged 65, (**d**) patient aged 71.

We used attention rollout to examine the basis for the age estimation of our hybrid model, focusing on the specific areas that the model considers. Dentition development is often considered an important factor in age estimation in young children (Fig. 5a). One noteworthy aspect was that the focus was placed more on the mandible than the maxilla, which is thought to be because mandible is freer from overlapping adjacent structures. For individuals in their late teens to early twenties, the focus of age estimation was on the second and third molars of both the maxilla and mandible (Fig. 5b). This is believed to be owing to distinct changes in the development of these teeth during this period. In older patients, age estimation is primarily based on the overall alveolar bone structure, and age-related or periodontal-induced alveolar bone loss appears to be a significant factor in determining age (Fig. 5c,d). Thus, the proposed model evidently uses logical and important factors rather than relying on unclear elements as the basis for age estimation. Creating datasets by extracting regions of interest (ROIs) for age-related anatomical structures while taking these logical and important factors into account can help develop more effective age estimation models.

In this experiment, only Korean data was used, but in the future, we plan to collect data regardless of race to design a model that is robust to external factors. In addition, future research directions are to solve the problem of non-uniformity in feature space that may occur during the data collection process.

## Conclusion

The proposed age estimation model designed using the hybrid method of the ResNet50 and ViT models exhibited better performance in predicting age by displaying better performance than those using ResNet50 or ViT, respectively. We expect this model to perform better and be used effectively in the clinical field.

## Data Availability

The data from this study can be made available, if required, within the regulation boundaries for data protection.
